# *Salmonella* identification from foods in eight hours: A prototype study with *Salmonella* Typhimurium

**Published:** 2012-03

**Authors:** A Koluman, G Celik, T Unlu

**Affiliations:** 1 National Food Reference Laboratory, Ankara, Republic of Turkiye; 2 Turkish Atomic Energy Authority, Saraykoy Nuclear Research and Training Center Ankara, Republic of Turkiye; 3 Ankara University, Veterinary Faculty, Dept. Microbiology, Ankara, Republic of Turkiye

**Keywords:** FTIR, IMS, *Salmonella*, PCR, Meat

## Abstract

**Background and Objectives:**

The significant rise in food borne infections is mainly caused by *Campylobacter* spp., *Salmonella* serovars and Verotoxigenic *Escherichia coli*. As the emerging food borne pathogens cause disease, more studies have been conducted for rapid detection of these pathogens. The combination of immunomagnetic separation and polymerase chain reaction (IMS-PCR) is the most accurate and rapid test preferred by almost every researcher. Fourier Transform Infrared Spectroscopy (FTIR) is preferred for being a new, user friendly and rapid technique in microbiological analyses. The main aim of this study is to detect application of IMS-FTIR for Salmonella identification from foods in a short time with a higher sensitivity.

**Materials and Methods:**

Conventional Culture Technique (CC), IMS-CC, IMS-PCR and IMS-FTIR techniques were compared with each other for rapid detection in artificially contaminated minced beef with *Salmonella* Typhimurium, as of the 2^nd^, 4^th^ and 8^th^ hours of contamination. The method was evaluated in different food matrices and sensitivity, specifity and overall recovery was calculated.

**Results:**

The results indicate that IMS-FTIR can detect *S*. Typhimurium as of the 8^th^ hour with sensitivity of 95.6667, accuracy of 91.69329, false positive ratio of 0.04333 and overall recovery of 95.66%.

**Conclusion:**

It can be suggested that the IMS-FTIR method is capable of detecting *S*.Typhimurium in a short time with lower cost.

## INTRODUCTION

Various techniques were developed for rapid identification of *Salmonella* serovars which has crucial role among food borne infections and intoxications. Immunomagnetic seperation (IMS) technique, among these techniques, has been used in publications where many studies were conducted by using various materials (1–9). IMS technique is of importance as it is both specific and sensitivity to *Salmonella* serovars and has advantages in the isolation process (9).

Fourier Transform Infrared Spectroscopy (FTIR) divides infrared signal consisting of a number of frequencies and shows each of them with their intensity by using Mathematical Fourier Transform Method. Despite the fact that the mentioned device is usually used with the purpose of identifying chemical materials, recent studies have underlined that various moves pertaining to bonds formed by elements found at the surface of bacteria with each other generates different reflections which are unique to each bacterium and finger prints of bacteria can be identified (10).

**Fig. 1:**
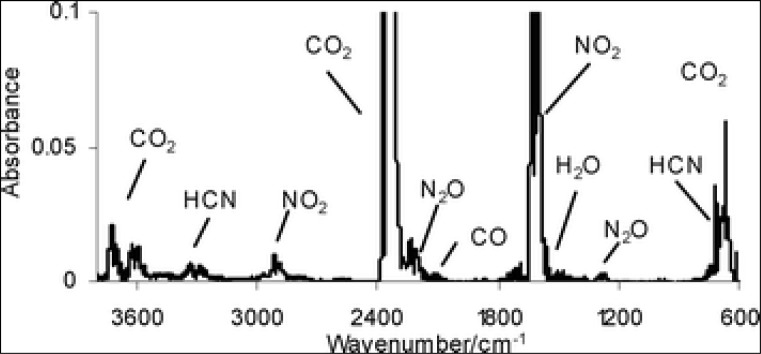
Peaks observed around bonds which are claimed to affect the spectrum (Naumann et al., 2006).

The fact that the device provides results in 15–45 seconds and performs Cluster analysis among the data it obtains, without requiring any consumable material resulted in intensive studies conducted by microbiologists all around the world. The studies have been conducted taking into account not only pathogen bacteria but also endospore forming bacteria, microorganisms producing hydrogen, lactic acid bacteria, yeasts, moulds and parasite eggs (11). Likewise, since the device can identify chemicals, it plays an active role in proteomics studies (12). The device mixes laser and infra-red rays proportional to each other and generates a spectrum depending on the reflection stemming from movements of molecular bond on the surface of the sample (13). Obtained spectrum are different for each product and example. Transformation in spectra differs depending not only on the kinds or species but also even on serotype. Spectra data pertaining to all bacteria produced under standard conditions are same. The bacteria can easily be defined in library where these standard finger prints exist. Seltmann et al. were the first to apply FTIR method in salmonella extract with the aim of rapid detection (14). It was shown that not only bacteria colony but also bacteria existing in homogenous biofilms could be identified by applying FTIR (15). Peaks observed around bonds which are claimed to affect the spectrum obtained from bacteria are shown in Fig. 1.

IMS-FTIR method has been used in limited number of studies. In relevant studies, *Salmonella* serovars which were smeared on chicken samples for experimental purposes (n = 25) were obtained by using IMS method and assessed in FTIR by making cluster analysis considering bacterial phenotypic determinants. Isolation of *Salmonella* serovars which were inoculated in the study was set as 84% and this ratio was reported to be insufficient since no other comparison was made. Given the data of the study, it was underlined that even one colony could be identified by using FTIR technology and thus, relations among all bacteria in one plate could be diagnosed (16).

This study was designed to evaluate the accuracy, ease of use and effectiveness of FTIR spectroscopy for *Salmonella* identification from food matrices. The aim of this study is to examine combination of FTIR and IMS considering identification of *Salmonella* Typhimurium. Classical Culture (CC), Immunomagnetic seperation –Polymerase Chain Reaction (IMS-PCR), IMS-CC and IMS-FTIR analyses were compared in terms of recovery, time and economy. Additionally the method has been evaluated in 10 different food matrices for validation.

## MATERIAL AND METHOD

This study aims at using FTIR technique for rapid detection. To this end, conventional cultural technique and IMS were applied simultaneously on minced beef samples contaminated by different bacteria. Bacteria obtained by using IMS were streaked on solid agar and identified by FTIR at phenotypic, PCR and genotypic level. Validation of the method is held with different 10 food matrices.

### Prototype study in minced meat samples

*M. longissimus dorsi* muscle was brought into laboratory under cold chain after the slaughter process. Sample brought under cold chain was trimmed under aseptic conditions and tendine fat and fascia were removed. The meat was minced using mincing machine. Then, it was put into sterile nylon bags in such a way that each bag has 250 gr portions. Samples were gamma irradiated with a dose of 10.0 kGy by using Gamacell. To this end, element Co^60^ was used, as radiation source, in Gamacell established with activated 10568 Ci by firm ISSLEDOVATELJ on 6 April 1994. Samples were kept at +4°C following the irradiation.

### Preparation of strains

Each strain for which lists are provided under Table 1 left in incubation in Brain Heart Infusion (BHI, CM0225, Oxoid, England) broth at 37 °C for 24 hours. Decimal dilutions were prepared from each suspension for all strains and Plate Count Agar (PCA, CM0325 Oxoid, England) was planted. After determining strenght of each strain, new decimal dilutions were prepared in such a way that each unit forms 9.0×10^3^ colony in millilitre (cfu/ml) and 1 ml from each strain was put into sterilized tubes and mixture of bacterial inoculum was prepared.

**Table 1:** Strains used in artificial contaminations.

*S*. Typhimurium	*Klebsiella pneumoniae*
(ATCC 14028, Oxoid, England)	(ATCC 13883, Oxoid, England)
*Pseudomonas aeruginosa*	*Escherichia coli*
(ATCC 10145, Oxoid, England)	(ATCC 10536, Oxoid, England)
*Citrobacter freundii*	*Proteus mirablis*
(ATCC 43864, Oxoid, England)	(ATCC 43071,RSHS Cultural collection, Turkey)

### Artificial contamination of minced meat samples

1 ml was taken from bacterial inoculum and this was added to samples that were divided into 25gr parts in filtered sterile sampling bags in biosafety cabinet (Airegard 201, Nuair, USA). All samples were added 225ml of buffered peptone water and the final contamination value was set approximately as 3.0×103 cfu/g after homogenised with sterile spatules and further in stomacher (Bagmixer 400VW, France). Five samples were used to form a batch under this study. Each batch was analysed with four different analyses. Mentioned analyses were made at different times. Accordingly, time of the first analysis is considered as hour 0. Analyses were made in following 2nd, 4th and 8th hours and examined with different isolation and identification methods discussed under this study.

### Timing of the analyses

At hours 0, 2, 4 and 8 of incubation respectively, conventional culture technique and IMS transaction were studied simultaneously in all groups. Microorganisms obtained with IMS were shifted into BPLS agar for further levels and FTIR and PCR were used.

### Number of analyses

Calculation of numbers of analysis = 5 batches (5 samples in each batch) ×4 methods ×4 different sampling time

### Analyses

**Fig. 2:**
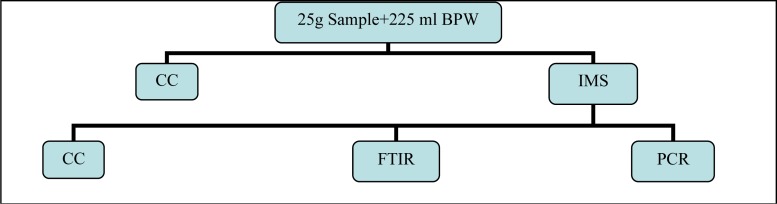
Implementation diagram pertaining to process flow.

Following homogenization, IMS transaction was made and sample kept for non selective enrichment. Remained parts were left in incubation at 37°C for non-selective pre-enrichment. Analyses were made from homogenizates left in incubation at hours 2, 4 and 8 of incubation. Implementation diagram pertaining to process flow is indicated in Fig. 2.

Homogenizates left in incubation was put into Rappaport Vassiliadis Broth for use of Clasical Culture Technique (CC). At the same time IMS process started and upon the completion of IMS, it was diluted with 100 µl sterilized physiological salty water and put in ultrasonic bath. It was ensured in ultrasonic bath that beads were separated from *S*. *Typhimurium*. 20 µl sample taken from here was evaluated in FTIR. 30 µl sample taken from the part remained after IMS transaction was subjected to DNA extraction and PCR process was carried out. 50 µl was taken from the part remained after suspension and CC technique was applied to it with Brillant Green Agar (BPLS, CM0263, Oxoid, England) streak. Analyses were repeated at 2nd, 4th and 8th hour. Methods applied in all analyses are provided in detail below.

### Conventional culture technique (CC)

*Salmonella* serovars were isolated in line with the method stated in ISO 6579 (17). To this end, 0,1 ml was taken from enrichment prepared in BPW and transferred into Rappaport Vassiliadis broth (RP, CM0669, Oxoid, England) and incubated at 43°C for 18 hours. 1 loopful was taken from selective enrichment and planted after streaked on to BPLS Agar, and then it was left in incubation at 37°C for 24 hours. *Salmonella* serovars formed pink transparent colonies. All colonies in suspect of *Salmonella* were identified with Microbact 12 E (Oxoid, England) rapid identification kits for further tests. At the same time, during each sampling, plantations were made in order to reveal total contamination level stemming from *Salmonella* and other bacteria in pre-enrichment.

### Immunomagnetic separation (IMS)

IMS transaction was executed with manual method as stated in prospectus of kit by Invitrogen by using Dynal anti-salmonella beads. Following IMS, all samples were kept in ultrasonic water bath (Clifton, MU22, England) at 37°C for 5 minutes with a view to ensuring separation from Dyna-Beads.

### Fourier transform infrared spectroscopy (FTIR)

FTIR device accessorized with Bruker bacteria identifier supported by OPUS software (Bruker, USA) with the aim of Fourier Transform Infrared Spectroscopy evaluated the spectra with wave length of Middle Infra Red (400 cm^−1^ and 4000 cm^−1^). Generated spectrum was matched with OPUS Lab Ident Program and existing library. To this end, IMS suspension was kept in Ultrasonic Bath for 5 minutes and was prepared and evaluated for spectra in such a way that is defined by OPUS in instructions for use. Accordingly, FTIR Bacteria Identifier (Bruker, HTS-XT, Tensor 27,) was diluted in 35 µl sterilized ultra pure water by taking single colony in sample preparation protocol and 30 µl from this mixture was transferred to ZnSe plates. Plates were dried in vacuum incubator under 500 mbar pressure at 25°C in such a way that a homogeneous film was obtained and they were evaluated for spectra in FTIR device in 4 hours. No dilution is made in suspensions but evaluated for spectra directly (18). Our protocol showed difference with the method given above. We used 20 µl of samples taken from IMS transactions.

### Polymerase chain reaction (PCR)

All DNA extractions were made with Invitrogen Purelink Genomic DNA Extraction Kit. PCR analyses were made in accordance with the method stated in Jenikova et al. on gene *inv*A (19). Primers used for *Salmonella* Typhimurium in line with relevant method are invA1 3′ACA GTG CTC GTT TAC GAC CTG AAT5′and invA2 5′AGA CGA CTG GTA CTG ATC GAT AAT 3′.

PCR Mastermix mixture: The study is made on 50 µl total volume. Accordingly, 5 µl 10× PCR amplification buffer (Metis, Turkey), 1.5 mM MgCl2, 200 µM dNTP (Metis, Turkey), 1 µM from each primary couple, 1.25 U Taq Polymerase (Promega, USA), 5 µl DNA template were added and prepared by adding ultra pure water in a way that total volume would be 50 µl. PCR cycles (Roche Light Cycler 24, Germany) are composed of 32 cycles in total. PCR was applied for 35 seconds at 94°C for denaturation, for 35 seconds at 56°C for binding, for 200 seconds at 72°C for extension. Following each cycle, a piece of DNA was denatured again at 94°C for 90 seconds and following last cycle extension was made at 72°C fore 12 minutes. PCR products were electrophoresed in agarose gel to which 1.2% ethidium bromide was added under 100-V current for 1 hour and band observed in 244 bp was accepted positive in gel imaging system (Geliance 200 Gel Imaging System, Perkin Elmer, USA).

### Validation of method with different food matrices

Ten different food matrices including; Powdered infant formula (PIF), Milk (MI), Minced Beef (MB), Cheddar Cheese (CH), Lettuce leaves (LT), Mayonnaise (MY), Ice cream (IC), Chicken Schnitzels (CS), Salami (SL) and Black pepper (BP) were spiked with *S*.Typhimurium at low, mid and high levels. All samples were spiked with *S*.Typhimurium at determined levels and IMS-FTIR was performed at 8^th^ hour of spiking. Spiking levels and results are given in Table 2.

**Table 2:** Validation study matrices and contamination levels.

Matrices	Number of samples spiked		

	Low (8×10^1^ cfu/ml)	Mid (1.1×10^2^ cfu/ml)	High (4.3×10^3^ cfu/ml)
Powdered infant formula (PIF)	10	10	10
Milk (MI)	10	10	10
Minced Beef (MB)	10	10	10
Cheddar Cheese (CH)	10	10	10
Lettuce leaves (LT)	10	10	10
Mayonnaise (MY)	10	10	10
Ice cream (IC)	10	10	10
Chicken Schnitzels (CS)	10	10	10
Salami (SL)	10	10	10
Black pepper (BP)	10	10	10
**TOTAL**	**100**	**100**	**100**

## RESULTS

**Fig. 3:**
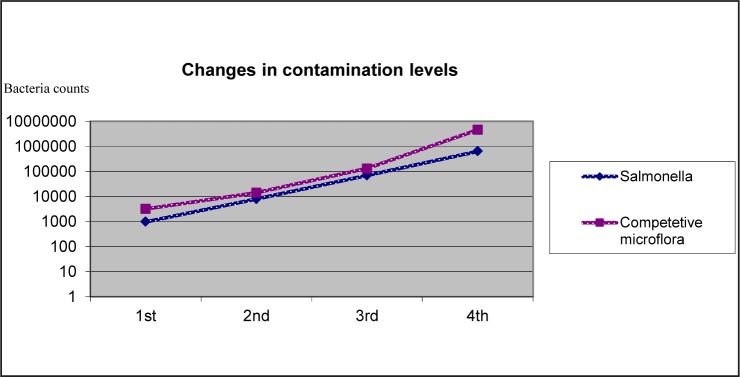
Time-dependant change in contamination level in *Salmonella* and other microflora.

In this study, recovery of *S*.Typhimurium carried out in 271 of 400 (67.75%) analyzes. The ratio of recovery of *S*.Typhimurium by analysis of CC, IMSCC, IMS-FTIR and IMS-PCR are 72, 77, 76 and 46%, respectively. During each sampling, *Salmonella* counts were observed, logarithmic increase may be observed with regard to bacterium. Relevant data is generated in Fig. 3.

**Fig. 4:**
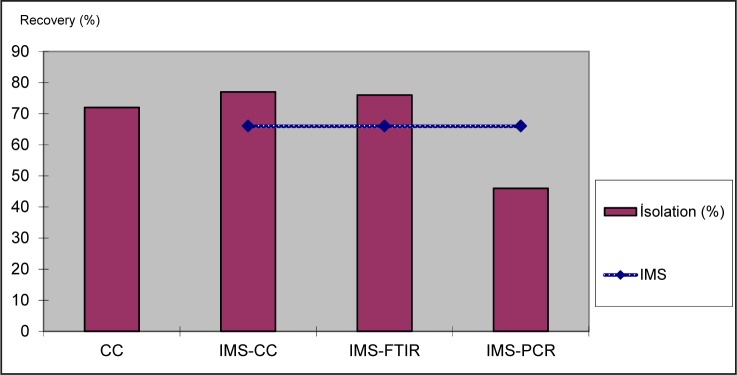
*S. Typhimurium* recovery ratios with different methods used in the study.

*S*. *Typhimurium* smeared with artificial contamination could be recovered in 199 (66.33%) out of 300 analyses where IMS method was applied. Isolation ratios (%) and *S*.Typhimurium recovery ratios (%) are shown in Fig. 4.

**Fig. 5:**
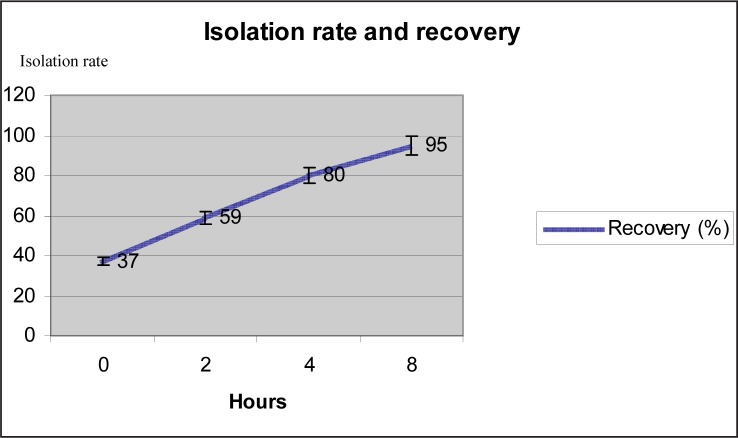
Recovery success determined by sampling time.

Analyses were held in different time periods under this study. Artificially contaminated samples were left in incubation at 37°C for 2 hours following contamination and upon the completion of mentioned two hours after first analysis were made. Time of the first analysis was considered as Hour 0. Then, analyses were made at 2^nd^, 4^th^ and 8^th^ hour by using different methods. Accordingly, *S*.Typhimurium smeared with artificial contamination could be recovered in 37 (37%) out of 100 analyses at hour 0, in 59 (59%) out of 100 analyses at 2^nd^ hour, in 80 (80%) out of 100 analyses at 4^th^ hour, and 95 (95%) out of 100 analyses at 8^th^ hour. Recovery success as per time is shown in Fig. 5.

**Fig. 6:**
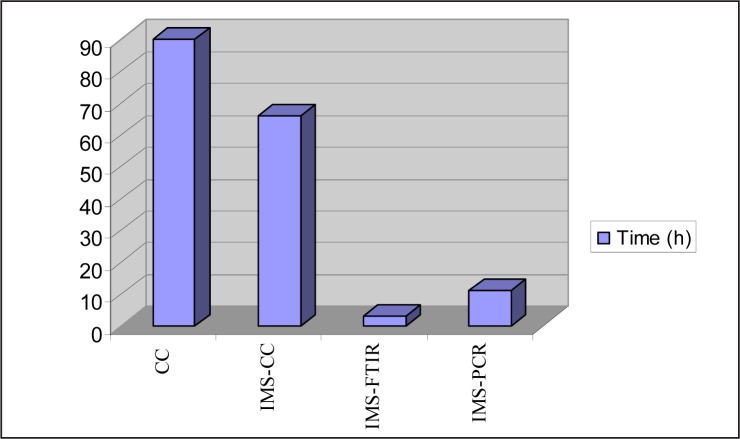
Change depending on elapsed time for each analysis type.

Passed time until complete analysis result was obtained with regard to 4 separate isolation and identification methods which are discussed under this study is as follows: Analyses took 90.6 hours by using conventional culture technique, 65.6 hours by using IMS Conventional Culture technique, 3.2 hours by using IMS-FTIR, 8.2 hours by using IMS-PCR. Change depending on elapsed time for each analysis type is shown in Fig. 6.

**Fig. 7:**
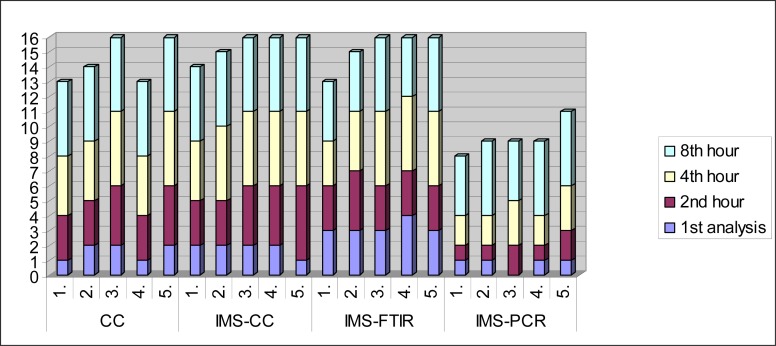
Comparison of all methods in terms of time and number of samples.

In calculations of data mentioned; pre-enrichment lasted 24 hours in average for CC, 18 hours for selective pre-enrichment, 24 hours for selective feed-lot, 24±12 hours for rapid detection system, 24 hours in average for IMS-CC and 2±0.5 hours in average for IMS. Likewise, considering IMSFTIR, IMS took 2±0.5 hours in average and the samples taken from here were put into ultrasonic bath for 5 minutes, vacuum was dried in incubator for 20 minutes and the spectrum was evaluated in FTIR device in 35 seconds. Average periods in IMSPCR process are as follows for each level; IMS lasted for 2±0.5 hours, DNA extraction for 1.4 hour, PCR cycles for 3.2 hours and electrophoresis on gel for 1 hour and no considerable time was spent for imaging. *K.pneumonia, P.aeruginosa, E.coli* and *C.freundii* strains which were added while preparing sample prevent isolation by covering *S*.Typhimurium colonies in conventional culture technique, likewise in IMSConventional culture technique sugar fermentors formed yellow colonies in BPLS agar, which was thought to stemmed from sampling pertaining to other bacteria taken from pre-enrichment liquid while transferring it into analysis tube. Decrease in isolation and recovery reduction rate observed in all methods were deemed to probably arise from seconder microflora (no data was shown). PCR Gel data are not shown. Considering all results, IMS-FTIR and IMS-CC are the techniques where most successful recovery is observed. Comparison of all methods is given in Fig. 7.

Cost for one sample under examination was analyzed considering all techniques. Each examination cost was calculated as per relevant methods, electricity, water, workmanship and prices of devices were ignored. All costs were calculated in Euros for more easily comparable data. 1 Euro was accepted as 2 Turkish Liras (TL) exchange rates were accepted as 1: 2. This exchange rate was calculated from the average of last 3 years (2009–2012). According to assessment, considering only consumable material costs calculated for one sample are as follows: cost of CC is 6.5 €, cost of IMS-KK is 10 €, cost of IMSFTIR is 4.5 €, cost of IMS-PCR is 16 €. It is pointed out that only cost of IMS is 4.5 €. Cost of CC analyses is remarkably high since rapid identification test Microbact has been used.

Detection of matrices effect on analysis was held on 10 different matrices. A sum of 300 samples were spiked at different levels of *S*.Typhimurium and 287 of 300 samples (95.66%) were found to be positive with the method and 13 samples (4.3%) gave negative results. The results are given Table 3.

**Table 3:** Validating study results.

Matrices	Positive results	Negative results	Recovery (%)
Powdered infant formula (PIF)	29	1	96.66
Milk (MI)	28	2	93.33
Minced Beef (MB)	28	2	93.33
Cheddar Cheese (CH)	30	0	100
Lettuce leaves (LT)	29	1	96.66
Mayonnaise (MY)	27	3	90
Ice cream (IC)	28	2	93.33
Chicken Schnitzels (CS)	29	1	96.66
Salami (SL)	29	1	96.66
Black pepper (BP)	30	0	100
**TOTAL**	**287**	**13**	**95.66**

The relative sensitivity of the method is 95.6667, accuracy is 91.69329, false positive ratio determined as 0.04333 and overall recovery was calculated as 95.66% for the new method. Best recovery rates were recorded in CH and BP.

## DISCUSSION

*S*. *Typhimurium* smeared with artificial contamination could be recovered in 271 (67.75%) out of 400 analyses under this study. *S*.Typhimurium artificial contamination could be recovered in 72 (72%) out of 100 analyses examined using CC, in 77 (77%) out of 100 analyses examined by using IMS-CC, 76 (76%) out of 100 analyses examined by using IMS-FTIR, 46 (46%) out of 100 analyses examined by using IMS-PCR. Likewise, *S*.Typhimurium artificial contamination could be recovered in 199 (66.33%) out of 300 analyses examined by using IMS method.

In a study, *Salmonella* isolation was made in 100 different food samples and to this end, 68% and 54% isolation and identification was obtained using classical culture technique and PCR technique (20). In another study by Jeníková, *et al*., artificial contamination was made in various foods with *Salmonella* Enteritidis. These were tried to be detected with culture and IMSPCR and it was stated that isolation may decrease in IMS-PCR due to competitive microflora (19). Kumar et al., in a IMS-PCR study, added *Salmonella typhi* culture to red meat rinse samples at 105 cfu/ml level and reported that they could ensure isolation from all (21). Bennet et al. indicated, in a study they conducted, that remaining of food matrix could affect the result in PCR (22). In another study, Muller Kaufmann Tetrathionate Broth (MKTT), Rappaport Vassiliadis Broth (RPV), RPV-PCR, Selecta ELISA, Selecta-Conventional Culture (CC), Optima ELISA, Optima-CC, BAX, Selecta PCR and Selecta IMS PCR methods were compared and RPV and RPV-PCR was stated as the most successful method (23).

Probable reason for low levels of IMS-PCR results may be caused by heterogeneity of bacteria during sample preparation. Additionally, competitive microflora added for experimental purposes might directly be connected to beads. Furthermore, it is thought that food matrix residues may have decrease power of IMS. In a study *Salmonella* serovars smeared for experimental purposes (n = 25) were obtained with IMS and subjected to cluster analysis considering bacterial phenotypic determinants in FTIR (16). Isolation ratio of *Salmonella* serovars inoculated in the study was found to be 84 % and this was reported to be insufficient since no other methodical comparison was made. Study data underlined that even one single colony could be identified by using FTIR technology, and thus interrelations among all bacteria in a plate could be revealed. Isolation ratio generated in this study and the isolation ratios generated in our study are close. In the same study inoculation levels were higher than our inoculation levels. However, they made isolation after 24 hours. It is deemed that variability of data generated in our study might be caused by time of sampling (16).

We designed the study to determine the most accurate time for IMS-FTIR. Due to this analyses were made in different times in this study. As a result, general low ratios of isolation in all analyses drew attention, which is thought to arise from time-dependant study.

In a similar study, time-dependant analysis was made on red meat samples which were experimentally contaminated with *Salmonella enterica* (2^nd^, 4^th^, 8^th^, 12^th^, 24^th^ hours following incubation) and they reported that no efficient result could be obtained earlier than 4 hours. Possible reason for this is the attachment of bacterium to food at lag phase and it is hard to recover bacteria. In this respect, they stated that significant recovery could be achieved in isolation by adding detergent derivatives to dilution environment since it would facilitate taking bacteria away from food (24). They also expressed that result could be obtained as of 4th hour in present study, but the findings become clear at the end of 8th hour, which is earlier when compared with other methods. These findings coincide with data summarized above.

Elapsed time until complete analyzed results were obtained with 4 different isolation and identification methods discussed under this study are as follows: Analyses lasted 90.6 hours by using conventional culture technique, 65.6 hours by using IMS Conventional Culture technique, 3.2 hours by using IMS-FTIR, 8.2 hours by using IMS-PCR. There are studies pointing out that FTIR analyses are rapid (10). Davis et al. The bacteria were detectable after 6 h of culture enrichment during a sensitivity experiment with lower initial inoculum of 10^1^ cfu/g (29). Classified *Enterococcus faecium* isolates obtained from clinical samples by using Pulsed Field Gel Electrophoresis and FTIR. It was pointed out, at the end of the study, that FTIR spectroscopy method is rapid not only for bacteria identification but also for Cluster analysis. Oberreuter et al. stated that they could accurately identify all of 208 isolates (100%) composed of bacteria included in *Corynebacterineae* in 25 hours following incubation (25).

In another similar study Amiel et al. reported that they could accurately identify 100% of lactic acid bacteria, in terms of species, in 22 hours (26). Naumann stated that, with a view to identifying species of different bacteria with FTIR spectroscopy, all of them must form at least 5mm in diameter of colony (27). Researcher stated that *Staphylococcus* spp. became apparent in 18 hours. Accordingly, the researcher expressed that FTIR responds in 18 hours at the earliest unless the researcher uses additional method. In the study made with IMS-FTIR 24-hour pre-enrichment was tried (16).

Cost for individual sample under examination was analysed considering all techniques. While cost is calculated as per relevant methods, electricity, water, workmanship and prices of devices were ignored. It is thought that a cheaper cost chart may be obtained if assessments are made according to conventional culture technique. It is pointed out that only cost of IMS is 4.5 €. Against the raise in cost, it is credible in detection phase. It was shown in this study that since the process was made in FTIR analysis through performing a bacteria suspension in distilled water, no material was consumed. Thus no expenditure would be incurred other than cost of the device. Naumann et al. stated that the only cost in FTIR analyses is the device (28).

Davis et al. reported FTIR as a faster and reliable method (29). De Lamo-Castellvi et al. reported that *Salmonella* cells bound to immunomagnetic beads had distinctive and reproducible infrared spectra and allowed characterization of particular bacterial structures but interference signal from the beads in the fingerprint region prevented accurate differentiation at the serovar level (30).

## CONCLUSION

Due to free movement of global food sources and tourism boost, many food-borne infections and intoxications have increased considerably. In parallel with this, treatment expenditures increase and its impacts on economy become unbearable. Isolation and identification of *Salmonella* serovars are tough and time consuming. *Salmonella* serovars are found in various foods and have many serotypes. In light of the data presented in our study, IMS-FTIR combination will detect *Salmonella* contamination existing in a food in 4 hours at the earliest and in 8 hours at the latest. Thus, IMS-FTIR, which does not require additional material, will have an outstanding place considering that it saves money, time and labour.
